# Post-transplant cyclophosphamide *versus* anti-thymocyte globulin in allogeneic hematopoietic stem cell transplantation from unrelated donors: A systematic review and meta-analysis

**DOI:** 10.3389/fonc.2023.1071268

**Published:** 2023-02-16

**Authors:** Lu Tang, Zhigang Liu, Tao Li, Tian Dong, Qiuhui Wu, Ting Niu, Ting Liu, Jie Ji

**Affiliations:** ^1^ West China Hospital, Sichuan University, Chengdu, China; ^2^ Department of Hematology, West China Hospital, Sichuan University, Chengdu, China; ^3^ Clinical Trial Center, West China Hospital, Sichuan University, Chengdu, China; ^4^ Department of Orthopedics, Orthopedic Research Institute, West China Hospital, Sichuan University, Chengdu, China

**Keywords:** post-transplant cyclophosphamide, allogeneic hematopoietic stem cell transplantation, graft-versus-host disease, infectious complication, anti-thymocyte globulin, unrelated donors

## Abstract

**Background:**

Post-transplant cyclophosphamide (PTCy) and anti-thymocyte globulin (ATG) are both common graft-versus-host disease (GVHD) prophylaxis strategies in allo-HSCT from unrelated donors. However, no consensus has reached on which regimen is optimal. Although several studies concerning this topic exist, the outcomes of different studies still conflict with each other. Therefore, an overall comparison of the two regimens is urgently needed to help make informed clinical decisions.

**Methods:**

Studies comparing PTCy and ATG regimens in unrelated donor (UD) allo-HSCT were searched in four critical medical databases from inception to April 17, 2022. The primary outcome was grade II-IV aGVHD, grade III-IV aGVHD and chronic GVHD (cGVHD), and the secondary outcomes included overall survival (OS), relapse incidence (RI), non-relapse mortality (NRM), and several severe infectious complications. The quality of articles was assessed by the Newcastle-Ottawa scale (NOS), and data were extracted by two independent investigators and then analyzed by RevMan 5.4.

**Results:**

Six out of 1091 articles were eligible for this meta-analysis. Compared with the ATG regimen, prophylaxis based on PTCy achieved a lower incidence of grade II-IV aGVHD incidence (RR=0.68, 95% CI 0.50-0.93, *P=*0.010, *I* ^2 =^ 67%), grade III-IV aGVHD (RR=0.32, 95% CI 0.14-0.76, *P*=0.001, *I* ^2 =^ 75%), NRM (RR=0.67, 95% CI 0.53-0.84, *P*=0.17, *I* ^2 =^ 36%), EBV-related PTLD (RR=0.23, 95% CI 0.09-0.58, *P*=0.85, *I* ^2 =^ 0%) and better OS (RR=1.29, 95% CI 1.03-1.62, *P=*0.0001, *I* ^2 =^ 80%). The cGVHD, RI, CMV reactivation and BKV-related HC showed no significant difference between the two groups (RR=0.66, 95% CI 0.35-1.26, *P*<0.00001, *I* ^2 =^ 86%; RR=0.95, 95% CI 0.78-1.16, *P*=0.37, *I* ^2 =^ 7%; RR=0.89, 95% CI 0.63-1.24, *P*=0.07, *I* ^2 =^ 57%; RR=0.88, 95% CI 0.76-1.03, *P*=0.44, *I* ^2 =^ 0%).

**Conclusion:**

In the setting of unrelated donor allo-HSCT, prophylaxis based on PTCy can lower the incidence of grade II-IV aGVHD, grade III-IV aGVHD, NRM and EBV-related complication, achieve better OS compared to ATG-based regimen. And cGVHD, RI, CMV reactivation and BKV-related HC were comparable in the two groups.

## 1 Introduction

Allogeneic hematopoietic stem cell transplantation (allo-HSCT) is a well-established curative treatment for various hematologic malignancies that provides hematopoietic reconstruction as well as the graft-versus-leukemia (GVL) effect (1). However, acute GVHD (aGVHD) is the leading cause of poor prognosis and thus restrain the implementation of allo-HSCT. The risk of GVHD is associated with the degree of human leukocyte antigen (HLA) match (2, 3). Unrelated donors are the main alternative for patients who lack HLA matched siblings. Furthermore, the high incidence of GVHD and non-relapse mortality (NMR) are reported in unrelated donors, especially in the setting of mismatched unrelated donors (MMUDs).

For unrelated donors, anti-thymocyte globulin (ATG)-based protocols is recommended as the standard GVHD prophylaxis, in combination with calcineurin inhibitors, and either methotrexate (MTX) or mycophenolate mofetil (MMF), which has been proven efficiently overcome the HLA disparity (4, 5). Nevertheless, ATG-based regimen is associated with more infections, especially viral ones (6–8). Recent years, Luznik et al. introduced posttransplant cyclophosphamide (PTCy) into haploidentical transplantation and reported relatively low GVHD and NRM (9). Gradually, PTCy with or without other immunosuppressive agents were administrated in other transplantation settings, including matched sibling donor (MSD), matched unrelated donor (MUD) and even MMUD (9–11). In study of Battipaglia et al., PTCy was demonstrated more favorable in GVHD prophylaxis and likely to provide better outcomes in the long run (12).

Although both PTCy- and ATG-based regimens have displayed efficacy in aGVHD prophylaxis, no consensus on which protocol is more effective has been reached. Although several studies compare the two strategies in setting of unrelated donors, their results contradict each other. Therefore, a comprehensive comparison between these two regimens in unrelated donors is in urgent need. Moreover, not only the incidence of GVHD but also infection is one of the major causes of NRM after transplantation (13). Up to now, comparisons of infection complications between these two strategies are still lacking. Gao et al. have already performed a meta-analysis to compare PTCy and ATG as GVHD prophylaxis regimens in terms of OS, NRM, relapse incidence (RI), leukemia-free survival (LFS), cGVHD, and aGVHD (14). However, the previous comparison just simply pooled results from various donor types together, thus making the outcome less convincing. In order to minimize the confounding results caused by different donor types, we only included MUD and MMUD for analysis. Furthermore, with the studies and articles emerging in the last two years, a new comparison between PTCy and ATG is necessary.

Consequently, in this meta-analysis, we updated the previous work by adding several current articles, including several infectious complications, to the primary outcomes. We also restricted the donor type to unrelated donors (MUD and MMUD) to provide more specific and comprehensive evidence for clinical decision-making.

## 2 Methods

### 2.1 Search strategy and study selection

We systematically searched PubMed, Embase, Web of Science, and the Cochrane library to select relevant articles up to April 17, 2022. We applied no language restrictions. We used the following combined text and MeSH terms: “ATG,” “anti-thymocyte globulin,” “GVHD,” “graft-versus-host disease,” “PTCy,” “posttransplant cyclophosphamide,” and “allogeneic transplantation.” The complete search used for PubMed is provided in **Supplementary Data 1**. We considered all potentially eligible studies for analysis, irrespective of the primary outcome or language.

After excluding duplications, two independent investigators reviewed the study titles and abstracts. All studies that satisfied the inclusion criteria were retrieved for full-text assessment. Two investigators analyzed trials selected for detailed analysis and data extraction with an agreement value (κ) of 96.5%; a third investigator resolved disagreements.

### 2.2 Inclusion and exclusion criteria

The criteria for eligible articles were as follows: (1) original human study; (2) patients diagnosed with hematological malignancies receiving allo-HCT; (3) receive MUD or MMUD; (4) comparison between PTCy and ATG; and (5) at least one of the following outcomes: incidence of grade II-IV and III-IV aGVHD, cGVHD, OS, RI, NRM, CMV reactivation, BKV reactivation, and EBV reactivation.

Criteria for exclusion: (1) duplicate studies, conference reports, letters, or other articles without available full text; (2) cell or animal studies; (3) receive other donor types: haploidentical donor or matched sibling donors; and (4) studies using PTCy and ATG in the same group.

### 2.3 Data extraction and quality assessment

Data extracted from all eligible articles included the first author, year of publication, journal, sample size, number of patients in the PTCy and ATG groups, patient age, patient sex, graft source, disease type, donor type, conditioning regimen, and follow-up time, and all these characteristics are provided in **Supplementary Table 1**.

In addition, primary clinical outcomes (grade II-IV and III-IV aGVHD, cGVHD, overall survival (OS), relapse incidence (RI), non-relapse mortality (NRM)), and infections (cytomegalovirus (CMV) reactivation and CMV disease, Epstein−Barr virus (EBV)-related posttransplant lymphoproliferative disease (PTLD), BKV-related hemorrhagic cystitis (HC)) were independently extracted from each article for aggregation and analysis. Two authors worked separately on a quality assessment by the Newcastle−Ottawa Scale (NOS) according to the NOS system’s three components (selection, comparability, and outcome). The range of scores is from 0 to 9, and a study with a score of 5 or higher is considered high quality (**Supplementary Table 2** *).*

### 2.4 Statistical analysis

This meta-analysis was performed with Review Manager (RevMan) software version 5.4. A two-sided P value ≤ 0.05 was considered statistically significant. All outcomes were counted as dichotomous variables, and events and total numbers were aggregated to compare the efficacy between the PTCy and ATG groups. This method was also adopted in subgroup analysis. We graphically displayed the results by forest plot. Heterogeneity was checked by the chi-square-based *Q* test and *I* ^2^ statistic (*I* ^2^ > 50%, *P ≤* 0.1 indicated high heterogeneity). If heterogeneity was considered not obvious, a fixed-effect model was employed for calculation; otherwise, a random-effect model was adopted.

## 3 Results

### 3.1 Search results and characteristics of the studies

**Figure 1:**
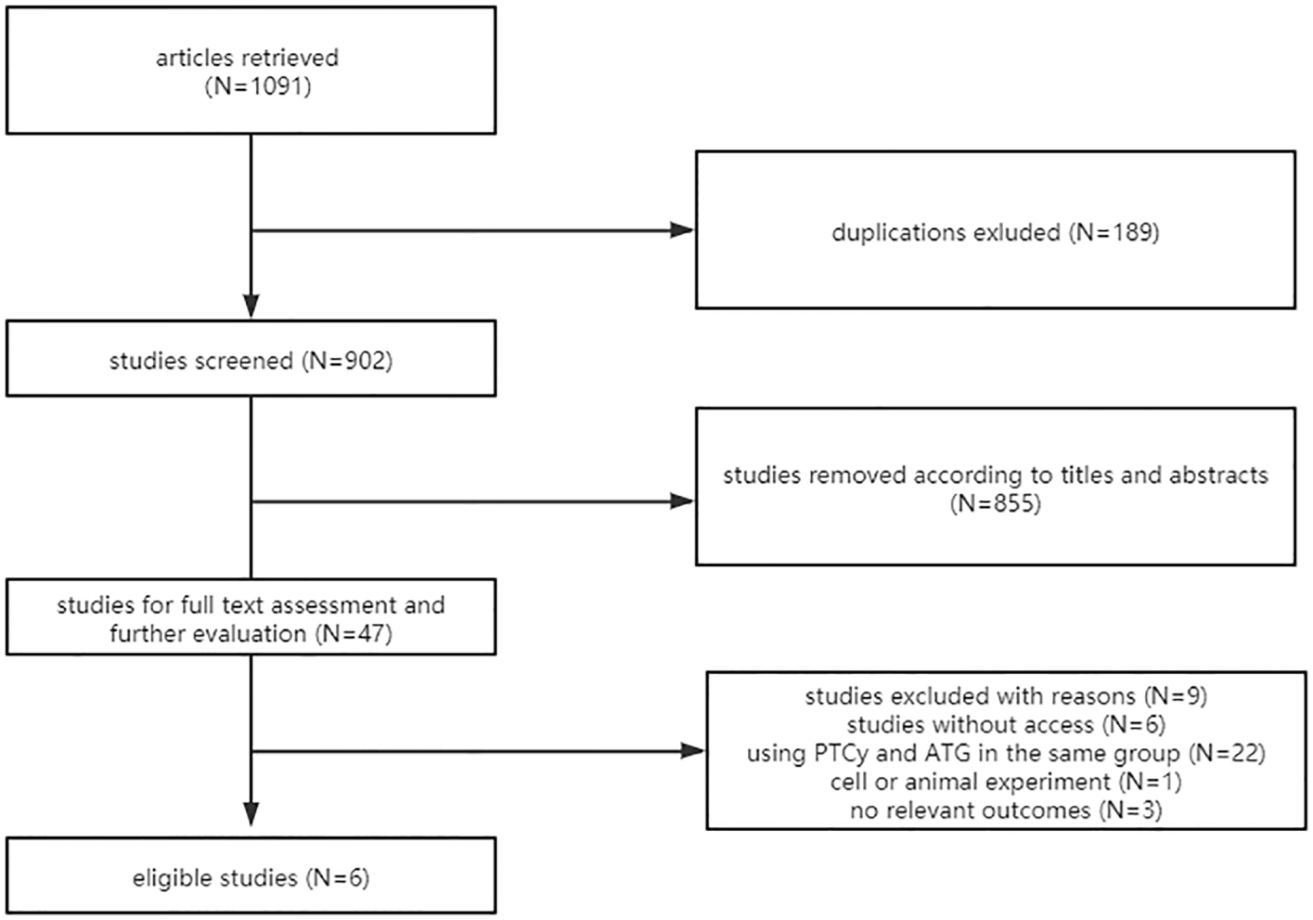
Flow diagram of study selection.

We identified 1091 articles, 206 from PubMed, 247 from Embase, 595 from Web of Science, and 43 from the Cochrane Library. After eliminating 189 duplications electronically, 902 potentially relevant publications were screened. Of these records, 855 citations were removed by reviewing the titles and abstracts. Full text or further details were retrieved for the remaining 47 citations. Finally, six eligible studies were included in the meta-analysis (12, 15–19). The flow diagram of the search and selection process is shown in **Figure 1**.

The six studies were published between 2016 and 2021 and were of relatively high quality (NOS scores higher than 5). All were retrospective studies, with a total of 2379 patients, of which 472 received PTCy and 1907 were treated with ATG.

The average age of patients in the six included studies ranged from 31 to 62. The median follow-up time varied substantially between groups, namely, 12 to 26 months in the PTCy group and 17 to 78 months in the ATG group. Acute myeloid leukemia (AML) and acute lymphoblastic leukemia (ALL) are the most common diseases. Among these six studies, there were three studies whose number of male subjects outweighed the number of female ones, only one study has more female patients, while another two did not mention the female-to-male ratio. The PTCy group had 472 unrelated donors, including 297 (62.9%) matched unrelated donors (MUDs) and 175 (37.1%) mismatched unrelated donors (MMUDs); while the ATG group included 1607 (84.3%) MUDs and 300 (15.7%) MMUDs. Of all the patients, 1093 (45.9%) subjects received myeloablative conditioning (MAC), including 201 (18.4%) in the PTCy group and 892 (81.6%) in the ATG group. The remaining 1286 (54.1%) patients were treated with nonmyeloablative or reduced-intensity conditioning (RIC), with 271 (21.1%) in the PTCy group and 1015 (78.9%) in the ATG group. For the graft source, 43 (9.1%) in the PTCy group were transplanted with bone marrow (BM) and the rest 429 (90.9%) received peripheral blood (PB) stem cells. In the ATG group, the number of patients who chose BM or PB as the graft source was 165 (8.7%) and 1742 (91.3%), respectively.

### 3.2 Grade II-IV and III-IV aGVHD

**Figure 2:**
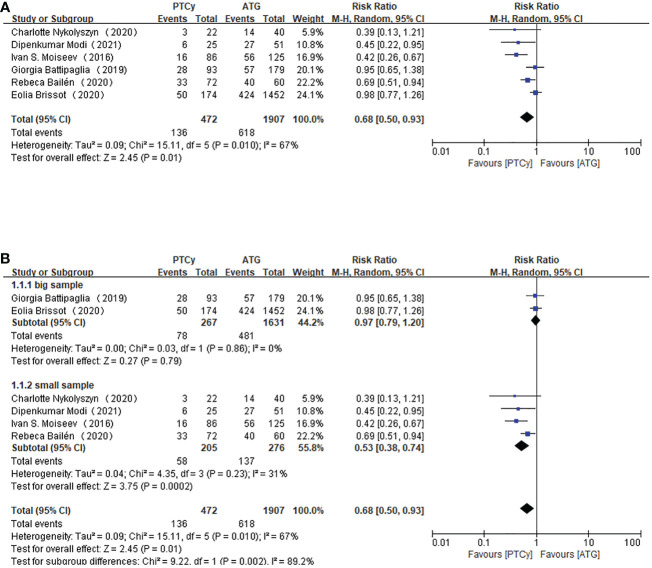
Forest plot of II-IV aGVHD **(A)** and Subgroup analysis of II-IV aGVHD according to the sample size **(B)**.

Six studies with 2379 patients all reported the incidence of grade II-IV aGVHD. We calculated each study’s events and total number as a dichotomous variable. Pooling the data of these studies showed that the PTCy has an advantage over ATG in grade II-IV aGVHD prevention. (RR=0.68, 95% CI 0.50-0.93, *P=*0.010, *I* ^2 =^ 67%; **Figure 2**). Considering the apparent heterogeneity, a sensitivity analysis and subgroup analysis were conducted. The studies were divided into two groups based on the sample size. Two studies were included into big sample group while the other four were in a small sample group. Patients in small sample size reported a lower incidence of grade II-IV aGVHD (RR=0.53, 95% CI 0.38-0.74, *P*=0.23, *I* ^2 =^ 31%; **Figure 2**) than large sample studies (RR=0.97, 95% CI 0.79-1.20, *P*=0.86, *I* ^2 =^ 0%; **Figure 2**), implying that the sample size of investigations might be the source of heterogeneity.

**Figure 3:**
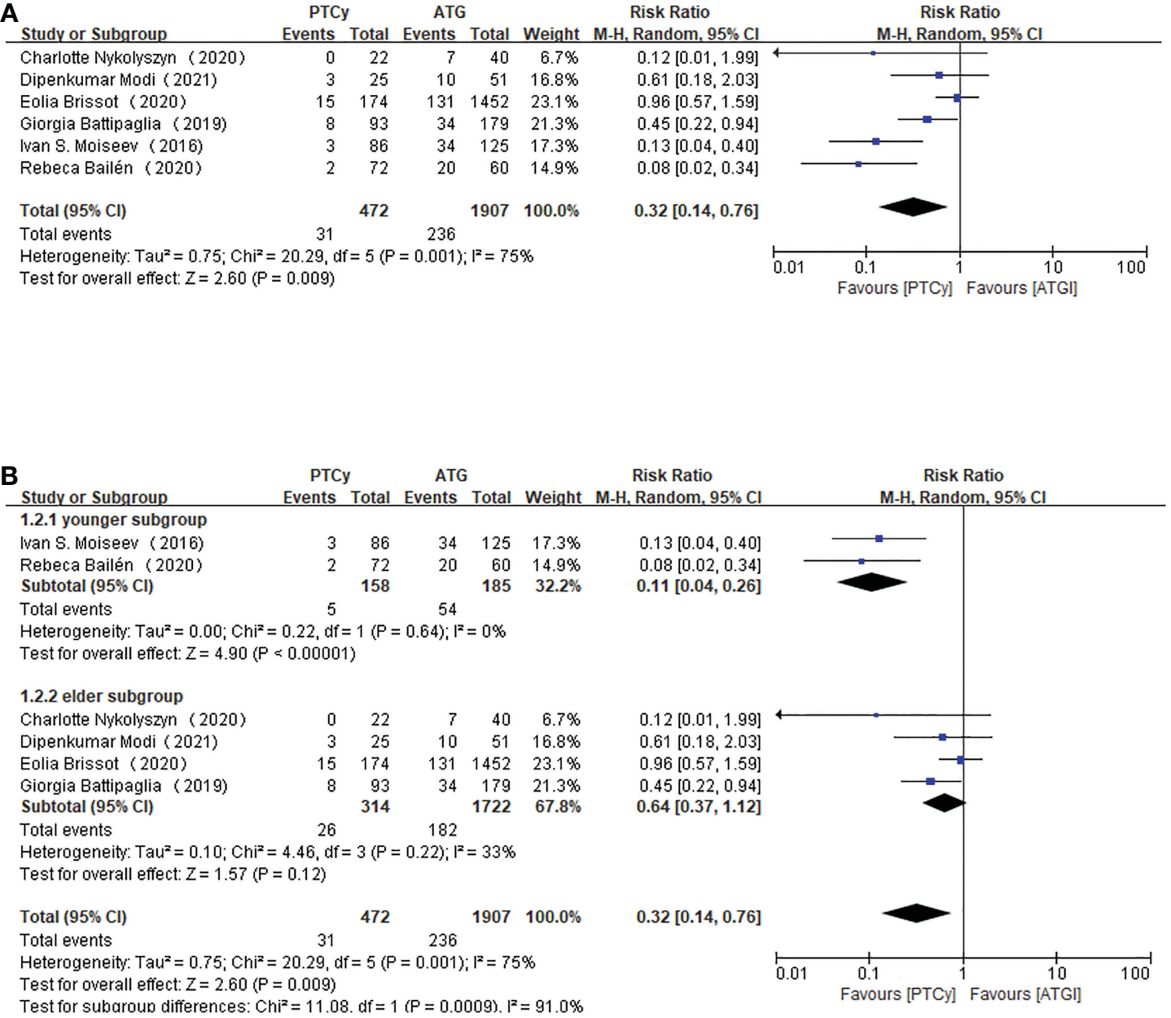
Forest plot of III-IV aGVHD **(A)** and subgroup analysis of III-IV aGVHD according to the age of the patients **(B)**.

Regarding grade III-IV aGVHD, all the included studies reported this outcome. The results demonstrated that prophylaxis based on PTCy was associated with a lower risk of developing grade III-IV aGVHD (RR=0.32, 95% CI 0.14-0.76, *P*=0.001, *I* ^2 =^ 75%; **Figure 3**). There was still heterogeneity, which could not be neglected. However, a sensitivity analysis showed that any separate study did not influence the heterogeneity. Therefore, subgroup analysis according to age was carried out. We took age 50 as the categorizing criterion. If the median age of both PTCy and ATG groups were lower than 50, the study would be categorized as younger subgroup; if not, the article would be placed in elder subgroup. The pooled results showed that the PTCy group tended to have a lower risk of III-IV aGVHD in the younger subgroup (RR=0.11, 95% CI 0.04-0.26, *P*=0.64, *I* ^2 =^ 0%; **Figure 3**) than elder subgroup (RR=0.64, 95% CI 0.37-1.12, *P*=0.22, *I* ^2 =^ 33%; **Figure 3**).

### 3.3 cGVHD

**Figure 4:**
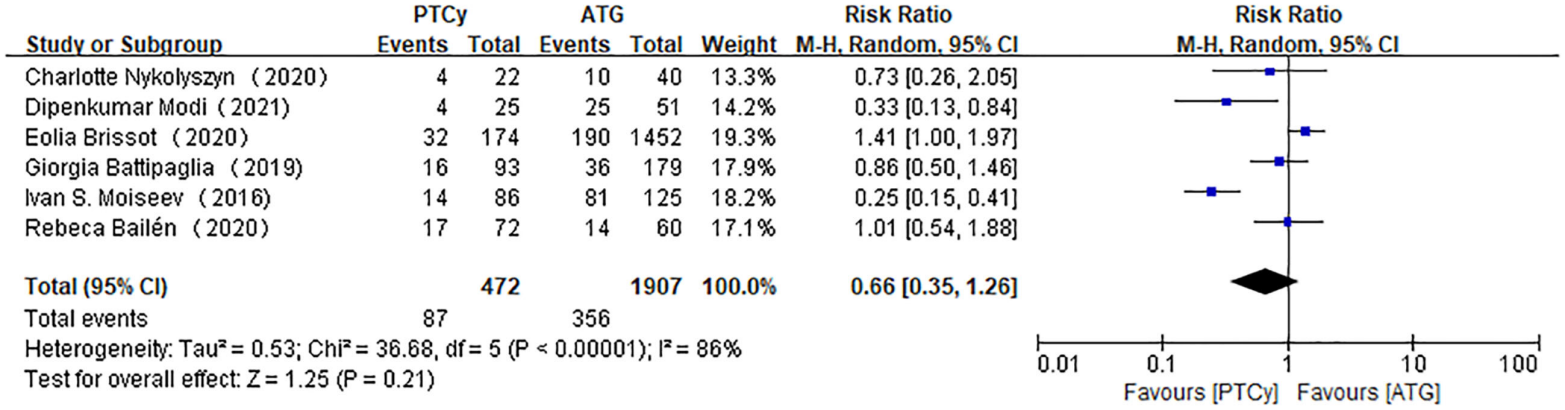
Forest plot of cGVHD.

Six studies were available for the analysis for moderate to severe cGVHD. The aggregated results showed no difference between PTCy and ATG regimens in cGVHD (RR=0.66, 95% CI 0.35-1.26, *P*<0.00001, *I* ^2 =^ 86%; **Figure 4**). A sensitivity analysis revealed that the obvious heterogeneity did not come from any included article. And subgroup analysis according to median age or the published year could not lower the heterogeneity.

### 3.4 OS

**Figure 5:**
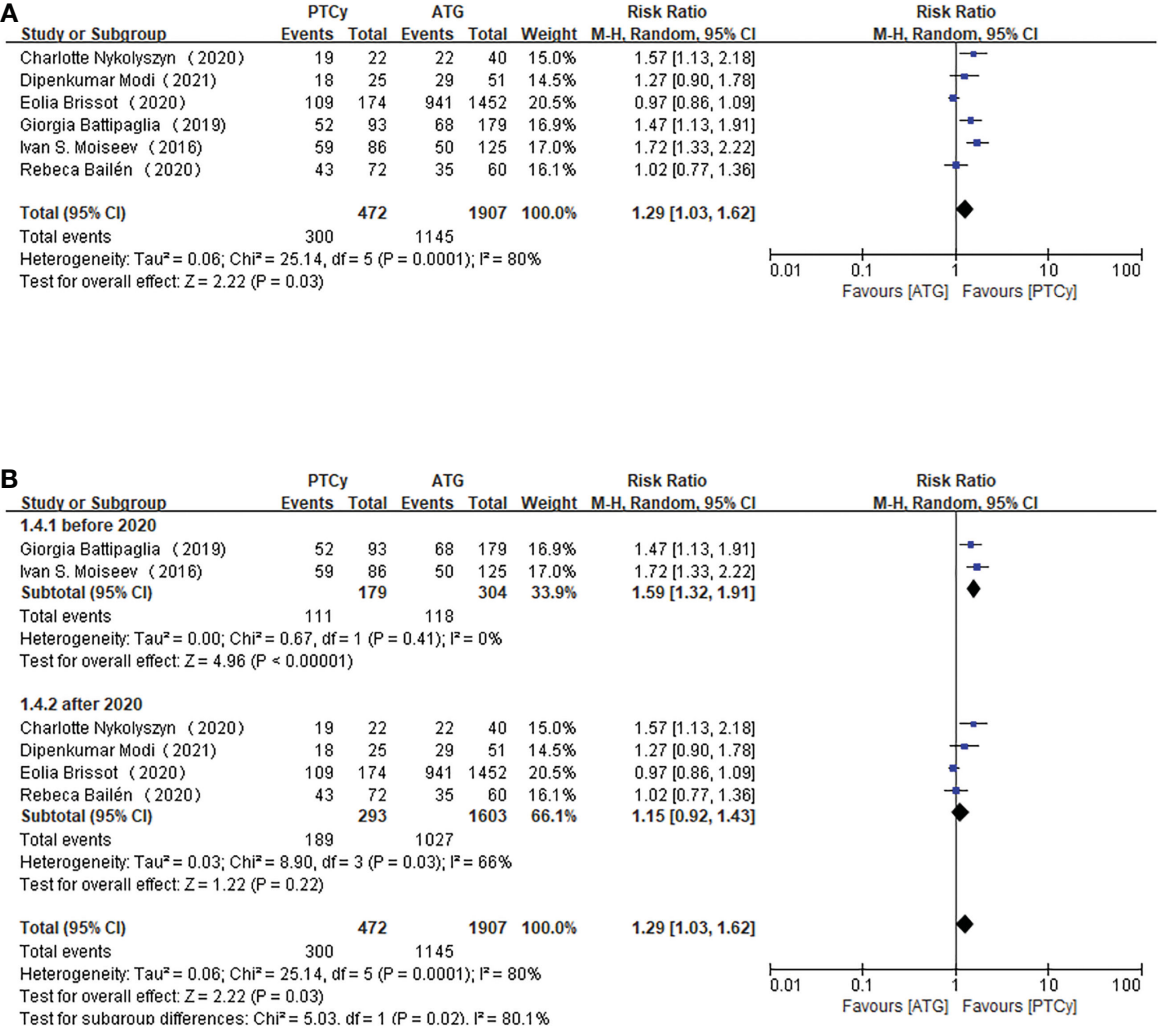
Forest plot of OS **(A)** and subgroup analysis of OS according to the published year **(B)**.

As for OS, according to the pooled RR, the use of PTCy was associated with a higher overall survival (RR=1.29, 95% CI 1.03-1.62, *P=*0.0001, *I* ^2 =^ 80%; **Figure 5**). Considering the obvious heterogeneity, a sensitivity analysis was applied to find the source of it. But ruling out any separate study did not significantly influence the result. However, subgroup analysis based on published year found that studies published before 2020 reported higher overall survival in PTCy group (RR=1.59, 95% CI 1.32-1.91, *P*=0.41, *I* ^2 =^ 0%; **Figure 5**).

### 3.5 RI and NRM

**Figure 6:**
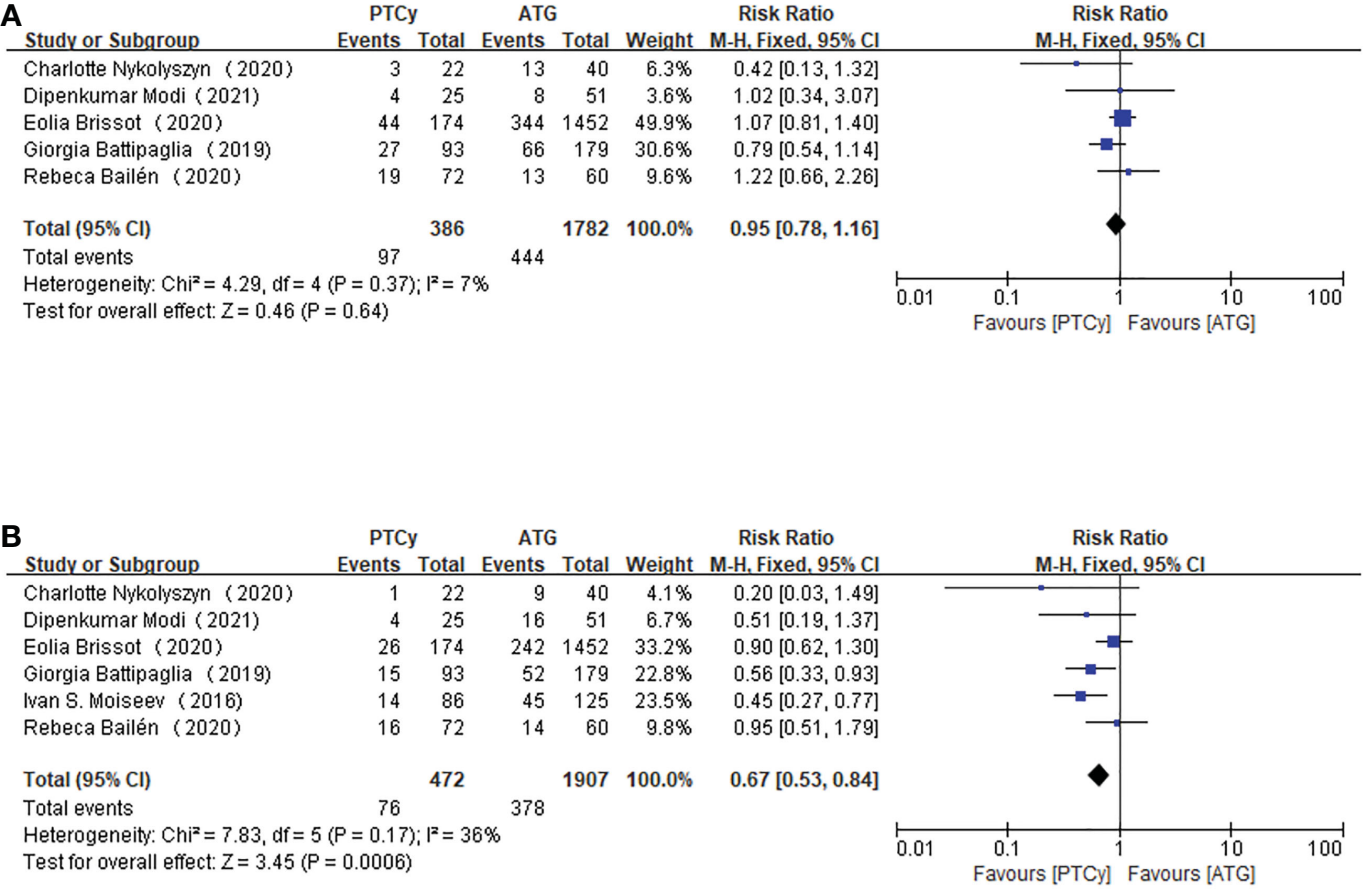
Forest plot of RI **(A)** and NRM **(B)**.

Among the six included studies, five of them reported the outcome of RI, while the rest one did not choose the relapse rate as primary outcome. There was no obvious heterogeneity among the included studies, therefore a fixed-effect model was used. The pooling data of RI showed no difference between the PTCy and ATG groups (RR=0.95, 95% CI 0.78-1.16, *P*=0.37, *I* ^2 =^ 7%; **Figure 6**).

For NRM, we gathered data from 2379 patients in all studies and adopted a fixed-effect model, which showed that, compared with ATG group, patients treated with PTCy is associated with lower NRM (RR=0.67, 95% CI 0.53-0.84, *P*=0.17, *I* ^2 =^ 36%; **Figure 6**). And the result was statistically significant as well.

### 3.6 Infectious complication

**Figure 7:**
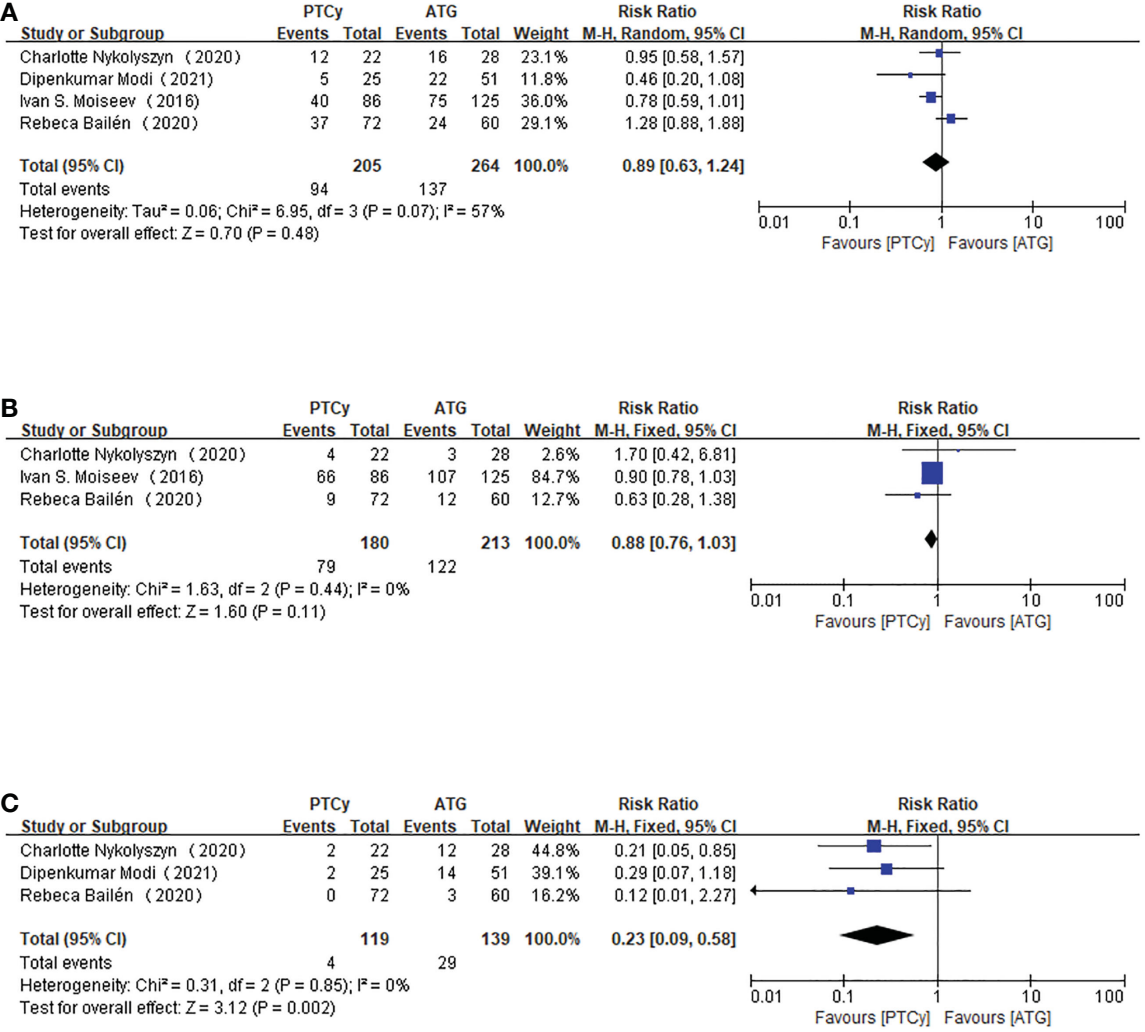
Forest plot of CMV reactivation (CMV disease) **(A)**, BKV-related HC **(B)** and EBV-related PTLD **(C)**.

The related data on infectious complications can be found in four articles. Four studies reported incidences of CMV reactivation and CMV disease. However, only three of them reported EBV-related PTLD and BKV-related HC. According to the aggregated RR value, we found that there was no significant difference in CMV reactivation and CMV disease between the PTCy group and ATG group (RR=0.89, 95% CI 0.63-1.24, *P*=0.07, *I* ^2 =^ 57%; **Figure 7**). Although apparent heterogeneity was observed, we did not conduct any sensitivity analysis or subgroup analysis due to the limited study data.

For BKV-related HC, we adopted the fixed-effect model because *I* ^2^<50%. The aggregated RR indicated that PTCy-based protocol and ATG-based prophylaxis were comparable in developing BKV-related HC. The heterogeneity was not significant (RR=0.88, 95% CI 0.76-1.03, *P*=0.44, *I* ^2 =^ 0%; **Figure 7**).

EBV-related PTLD was only reported in three articles, and further analysis showed that the heterogeneity was not apparent; therefore, a fixed-effect model was used to assess the outcome. The results indicated that adopting the PTCy regimen was associated with a lower chance of developing EBV-related PTLD disease (RR=0.23, 95% CI 0.09-0.58, *P*=0.85, *I* ^2 =^ 0%; **Figure 7**), and the result was statistically significant.

## 4 Discussion

Allo-HSCT is an essential treatment for patients with hematological malignancies. Nevertheless, the incidence of aGVHD remains the major obstacle to the full benefit of the transplant. Regimens based on PTCy or ATG were both used for aGVHD prophylaxis in setting of unrelated donors (9, 20–22). The main objective of this meta-analysis was to compare the two regimens in MUD and MMUD and determine which one provided better outcomes.

The results of this meta-analysis indicated that, compared with ATG, the administration of PTCy reduced the incidence of all grades aGVHD but did not alter the risk of developing cGVHD. This result of aGVHD was in line with many previous results, which showed that PTCy was more favorable for reducing the incidence of both grade II-IV and grade III-IV aGVHD (12, 16, 19, 23). The feasibility and efficacy of PTCy as a single agent for aGVHD prevention have been proven in a body of studies, especially when patients are transplanted with BM cells (9, 11). However, in peripheral blood stem cell (PBSC) graft settings, a single agent of Cy may not be enough to prevent severe aGVHD (24, 25). Considering that most of the patients in this meta-analysis received PTCy-based GVHD prophylaxis in the setting of PBSC and the incidence in aGVHD was acceptable, we speculated that other immunosuppressive agents used simultaneously might play a role. Carnevale-Schianca et al. used a combination of PTCy and tacrolimus-MMF to prevent GVHD in PBSC from HLA-matched donors and found that the incidence of aGVHD was hugely reduced (26). And low-dose ATG/PTCy combined with CsA/MMF as GvHD prophylaxis in MUD-PBSCT had promising activity (27). Additionally, the subgroup analysis in grade III-IV aGVHD based on the age of subjects illustrated that the administration of the PTCy agent might exert a better prophylactic effect in the younger subgroup. This can be easily understood because many adverse events after transplantation can be attributed to the confounding disadvantages of increasing age. The similar incidence of moderate to severe cGVHD in two regimens may be attributed to the cells targeted by ATG, not only donor T cells but also neutrophils, monocytes, NK- and B cells and non-immune cells such as endothelial cells, which are crucial in developing cGVHD (28, 29). However, all the results should be interpreted with caution for the high heterogeneity.

Concerning infections, the lower incidence of EBV-related PTLD and the comparable incidence of CMV reactivation and BKV-related HC are consistent with previous studies. Bailén et al. reported all these infectious complications between the two regimens, and the results demonstrated that the risk of EBV reactivation was higher in the ATG group. In contrast, CMV reactivation and HC were similar between groups (18). Moreover, Walker et al. and Finke et al. also found higher EBV reactivation in the ATG group in unrelated donor setting (20, 30). However, the incidence of BKV-related HC was considered lower in the PTCy group with statistical significance in the study of Ciurea et al., which disagreed with our findings (4). One possible explanation for the similar BKV-related HC in two regimens was that the low dose of ATG in the included studies. A higher dose of ATG is associated with a higher risk of infection or PTLD (20, 31) because ATG may negatively affect survival by increasing infection and relapse rate (18). But that should be interpreted with caution for the dose of ATG in several included studies was not available or not defined, thus limiting the possibility to explore its specific influence on the main transplantation outcomes. Moreover, this finding might also be related to insufficient infection data.

Despite the concern about the high relapse rate with the administration of PTCy in haplo-HSCT for hematologic diseases, which is up to 50% (23, 32). However, in this meta-analysis, no differences were observed between the two groups concerning RI. And PTCy-based prophylaxis even achieves better OS and NRM. The difference in NRM can be partially boiled down to the higher infection rate in ATG group, especially viral infection. At the same time, a matched-pair analysis performed by the ALWP of the EBMT showed that PTCy was associated with better LFS and GVHD-free relapse-free survival (GRFS) in patients undergoing 9/10 UD-HSCT (12). And lower NRM in PTCy group may contribute to improved OS.

Undeniably, there are several unavoidable limitations of this analysis. First, almost all included studies were retrospective studies rather than prospective randomized controlled trials, which may result in irremovable bias. Furthermore, the heterogeneity in several outcomes was notable, probably due to the unmeasured factors caused by the retrospective nature of this analysis. However, sensitivity and subgroup analyses were conducted to identify the potential source of heterogeneity.

Collectively, in the absence of prospective, randomized data, the results of this meta-analysis indicated that compared with ATG, the administration of PTCy demonstrated better prevention for grade II-IV and grade III-IV aGVHD, achieve better OS and NRM, and was associated with a lower incidence of EBV-related PTLD without increasing other risks. Therefore, using PTCy as GvHD prophylaxis may be considered an optimal strategy for the prevention of GVHD in patients undergoing UD-HSCT.

## Data availability statement

The original contributions presented in the study are included in the article/**Supplementary Material**. Further inquiries can be directed to the corresponding author.

## Author contributions

All authors contributed to the study conception and design. JJ had the idea for the article. Literature search and data analysis were performed by LT, ZL, and TOL. TD, QW, TN, and TL critically revised the work. All authors contributed to the article and approved the submitted version.
