# Supplementary material for: Post-transplant cyclophosphamide versus anti-thymocyte globulin in allogeneic hematopoietic stem cell transplantation from unrelated donors: A systematic review and meta-analysis
Source: Front Oncol. 2023 Feb 16;13:1071268. doi: 10.3389/fonc.2023.1071268 (PMC9978173; doi:10.3389/fonc.2023.1071268)
Supplement: Supplementary file 1 [file DataSheet_1.docx]

**Searching strategy in PubMed**

((((((((((("cyclophosphamide"[MeSH Terms] OR "cyclophosphamide"[All Fields] OR "cyclophosphamid"[All Fields] OR "cyclophosphamide s"[All Fields] OR "cyclophosphamides"[All Fields] OR ("cyclophosphamide"[MeSH Terms] OR "cyclophosphamide"[All Fields] OR "sendoxan"[All Fields]) OR ("cyclophosphamide"[MeSH Terms] OR "cyclophosphamide"[All Fields] OR "b 518"[All Fields]) OR ("cyclophosphamide"[MeSH Terms] OR "cyclophosphamide"[All Fields] OR "b 518"[All Fields]) OR ("cyclophosphamide"[MeSH Terms] OR "cyclophosphamide"[All Fields] OR "b518"[All Fields]) OR ("cyclophosphamide"[MeSH Terms] OR "cyclophosphamide"[All Fields] OR ("cyclophosphamide"[All Fields] AND "anhydrous"[All Fields]) OR "cyclophosphamide anhydrous"[All Fields]) OR ("cyclophosphamide"[MeSH Terms] OR "cyclophosphamide"[All Fields] OR "cyclophosphamid"[All Fields] OR "cyclophosphamide s"[All Fields] OR "cyclophosphamides"[All Fields])) AND "R"[All Fields]) AND ("isomerism"[MeSH Terms] OR "isomerism"[All Fields] OR "isomer"[All Fields] OR "isomers"[All Fields])) OR ("cyclophosphamide"[MeSH Terms] OR "cyclophosphamide"[All Fields] OR "cyclophosphamid"[All Fields] OR "cyclophosphamide s"[All Fields] OR "cyclophosphamides"[All Fields])) AND "S"[All Fields]) AND ("isomerism"[MeSH Terms] OR "isomerism"[All Fields] OR "isomer"[All Fields] OR "isomers"[All Fields])) OR ("cyclophosphamide"[MeSH Terms] OR "cyclophosphamide"[All Fields] OR "cytophosphane"[All Fields]) OR ("cyclophosphamide"[MeSH Terms] OR "cyclophosphamide"[All Fields] OR ("cyclophosphamide"[All Fields] AND "monohydrate"[All Fields]) OR "cyclophosphamide monohydrate"[All Fields]) OR ("cyclophosphamide"[MeSH Terms] OR "cyclophosphamide"[All Fields]) OR ("cyclophosphamide"[MeSH Terms] OR "cyclophosphamide"[All Fields] OR "cytoxan"[All Fields]) OR ("cyclophosphamide"[MeSH Terms] OR "cyclophosphamide"[All Fields] OR "endoxan"[All Fields]) OR ("cyclophosphamide"[MeSH Terms] OR "cyclophosphamide"[All Fields] OR "neosar"[All Fields]) OR ("cyclophosphamide"[MeSH Terms] OR "cyclophosphamide"[All Fields] OR "nsc 26271"[All Fields]) OR ("cyclophosphamide"[MeSH Terms] OR "cyclophosphamide"[All Fields] OR "nsc 26271"[All Fields]) OR ("cyclophosphamide"[MeSH Terms] OR "cyclophosphamide"[All Fields] OR "nsc26271"[All Fields]) OR ("cyclophosphamide"[MeSH Terms] OR "cyclophosphamide"[All Fields] OR "procytox"[All Fields]) ()) AND "2"[All Fields]) AND (("bis"[All Fields] AND "2-Chloroethyl"[All Fields]) AND ("amino"[All Fields] OR "aminos"[All Fields]))) AND ("tetrahydro-2H-1"[All Fields] AND "3"[All Fields] AND "2-oxazaphosphorine"[All Fields] AND "2-Oxide"[All Fields] AND ("monohydrate"[All Fields] OR "monohydrated"[All Fields] OR "monohydrates"[All Fields] OR "monohydration"[All Fields]))) OR ("cyclophosphamide"[MeSH Terms] OR "cyclophosphamide"[All Fields] OR "cyclophosphan"[All Fields] OR "cyclophosphane"[All Fields])) AND ((("antilymphocyte serum"[MeSH Terms] OR ("antilymphocyte"[All Fields] AND "serum"[All Fields]) OR "antilymphocyte serum"[All Fields] OR ("antilymphocyte"[All Fields] AND "serums"[All Fields]) OR "antilymphocyte serums"[All Fields] OR ("antilymphocyte serum"[MeSH Terms] OR ("antilymphocyte"[All Fields] AND "serum"[All Fields]) OR "antilymphocyte serum"[All Fields] OR ("serum"[All Fields] AND "antilymphocyte"[All Fields]) OR "serum antilymphocyte"[All Fields]) OR ("antilymphocyte serum"[MeSH Terms] OR ("antilymphocyte"[All Fields] AND "serum"[All Fields]) OR "antilymphocyte serum"[All Fields] OR ("serums"[All Fields] AND "antilymphocyte"[All Fields])) OR ("antilymphocyte serum"[MeSH Terms] OR ("antilymphocyte"[All Fields] AND "serum"[All Fields]) OR "antilymphocyte serum"[All Fields] OR ("antilymphocyte"[All Fields] AND "antibodies"[All Fields]) OR "antilymphocyte antibodies"[All Fields]) OR ("antilymphocyte serum"[MeSH Terms] OR ("antilymphocyte"[All Fields] AND "serum"[All Fields]) OR "antilymphocyte serum"[All Fields] OR ("antibodies"[All Fields] AND "antilymphocyte"[All Fields]) OR "antibodies antilymphocyte"[All Fields]) OR ("antilymphocyte serum"[MeSH Terms] OR ("antilymphocyte"[All Fields] AND "serum"[All Fields]) OR "antilymphocyte serum"[All Fields] OR ("antibody"[All Fields] AND "antilymphocyte"[All Fields]) OR "antibody antilymphocyte"[All Fields]) OR ("antilymphocyte serum"[MeSH Terms] OR ("antilymphocyte"[All Fields] AND "serum"[All Fields]) OR "antilymphocyte serum"[All Fields] OR ("antilymphocyte"[All Fields] AND "antibody"[All Fields]) OR "antilymphocyte antibody"[All Fields]) OR ("antilymphocyte serum"[MeSH Terms] OR ("antilymphocyte"[All Fields] AND "serum"[All Fields]) OR "antilymphocyte serum"[All Fields] OR ("antilymphocyte"[All Fields] AND "globulin"[All Fields]) OR "antilymphocyte globulin"[All Fields]) OR ("antilymphocyte serum"[MeSH Terms] OR ("antilymphocyte"[All Fields] AND "serum"[All Fields]) OR "antilymphocyte serum"[All Fields] OR ("antilymphocyte"[All Fields] AND "globulins"[All Fields]) OR "antilymphocyte globulins"[All Fields]) OR ("antilymphocyte serum"[MeSH Terms] OR ("antilymphocyte"[All Fields] AND "serum"[All Fields]) OR "antilymphocyte serum"[All Fields] OR ("globulin"[All Fields] AND "antilymphocyte"[All Fields]) OR "globulin antilymphocyte"[All Fields]) OR ("antilymphocyte serum"[MeSH Terms] OR ("antilymphocyte"[All Fields] AND "serum"[All Fields]) OR "antilymphocyte serum"[All Fields] OR ("globulins"[All Fields] AND "antilymphocyte"[All Fields])) OR ("antilymphocyte serum"[MeSH Terms] OR ("antilymphocyte"[All Fields] AND "serum"[All Fields]) OR "antilymphocyte serum"[All Fields] OR "pressimmune"[All Fields]) OR ("antilymphocyte serum"[MeSH Terms] OR ("antilymphocyte"[All Fields] AND "serum"[All Fields]) OR "antilymphocyte serum"[All Fields] OR "antithymoglobulin"[All Fields]) OR ("antilymphocyte serum"[MeSH Terms] OR ("antilymphocyte"[All Fields] AND "serum"[All Fields]) OR "antilymphocyte serum"[All Fields] OR "antithymoglobulins"[All Fields]) OR ("antilymphocyte serum"[MeSH Terms] OR ("antilymphocyte"[All Fields] AND "serum"[All Fields]) OR "antilymphocyte serum"[All Fields] OR ("antithymocyte"[All Fields] AND "globulin"[All Fields]) OR "antithymocyte globulin"[All Fields]) OR ("antilymphocyte serum"[MeSH Terms] OR ("antilymphocyte"[All Fields] AND "serum"[All Fields]) OR "antilymphocyte serum"[All Fields] OR ("antithymocyte"[All Fields] AND "globulins"[All Fields]) OR "antithymocyte globulins"[All Fields]) OR ("antilymphocyte serum"[MeSH Terms] OR ("antilymphocyte"[All Fields] AND "serum"[All Fields]) OR "antilymphocyte serum"[All Fields] OR ("globulin"[All Fields] AND "antithymocyte"[All Fields]) OR "globulin antithymocyte"[All Fields]) OR ("antilymphocyte serum"[MeSH Terms] OR ("antilymphocyte"[All Fields] AND "serum"[All Fields]) OR "antilymphocyte serum"[All Fields] OR ("globulins"[All Fields] AND "antithymocyte"[All Fields]) OR "globulins antithymocyte"[All Fields]) OR ("antilymphocyte serum"[MeSH Terms] OR ("antilymphocyte"[All Fields] AND "serum"[All Fields]) OR "antilymphocyte serum"[All Fields] OR ("lymphocyte"[All Fields] AND "immune"[All Fields] AND "globulin"[All Fields] AND "anti"[All Fields] AND "thymocyte"[All Fields] AND "globulin"[All Fields])) OR ("antilymphocyte serum"[MeSH Terms] OR ("antilymphocyte"[All Fields] AND "serum"[All Fields]) OR "antilymphocyte serum"[All Fields] OR ("lymphocyte"[All Fields] AND "immune"[All Fields] AND "globulin"[All Fields] AND "anti"[All Fields] AND "thymocyte"[All Fields] AND "globulin"[All Fields])) OR ("antilymphocyte serum"[MeSH Terms] OR ("antilymphocyte"[All Fields] AND "serum"[All Fields]) OR "antilymphocyte serum"[All Fields] OR ("anti"[All Fields] AND "thymocyte"[All Fields] AND "globulin"[All Fields]) OR "anti thymocyte globulin"[All Fields]) OR ("antilymphocyte serum"[MeSH Terms] OR ("antilymphocyte"[All Fields] AND "serum"[All Fields]) OR "antilymphocyte serum"[All Fields] OR ("anti"[All Fields] AND "thymocyte"[All Fields] AND "globulin"[All Fields]) OR "anti thymocyte globulin"[All Fields]) OR ("antilymphocyte serum"[MeSH Terms] OR ("antilymphocyte"[All Fields] AND "serum"[All Fields]) OR "antilymphocyte serum"[All Fields] OR ("anti"[All Fields] AND "thymocyte"[All Fields] AND "globulins"[All Fields]) OR "anti thymocyte globulins"[All Fields]) OR ("antilymphocyte serum"[MeSH Terms] OR ("antilymphocyte"[All Fields] AND "serum"[All Fields]) OR "antilymphocyte serum"[All Fields] OR ("globulin"[All Fields] AND "anti"[All Fields] AND "thymocyte"[All Fields]) OR "globulin anti thymocyte"[All Fields]) OR ("antilymphocyte serum"[MeSH Terms] OR ("antilymphocyte"[All Fields] AND "serum"[All Fields]) OR "antilymphocyte serum"[All Fields] OR ("globulins"[All Fields] AND "anti"[All Fields] AND "thymocyte"[All Fields])) OR ("antilymphocyte serum"[MeSH Terms] OR ("antilymphocyte"[All Fields] AND "serum"[All Fields]) OR "antilymphocyte serum"[All Fields] OR "atgam"[All Fields] OR ("lymphocyte"[All Fields] AND "immune"[All Fields] AND "globulin"[All Fields] AND "anti"[All Fields] AND "thymocyte"[All Fields] AND "globulin"[All Fields]) OR "lymphocyte immune globulin"[All Fields]) OR ("antilymphocyte serum"[MeSH Terms] OR ("antilymphocyte"[All Fields] AND "serum"[All Fields]) OR "antilymphocyte serum"[All Fields] OR ("lymphocyte"[All Fields] AND "immune"[All Fields] AND "globulin"[All Fields] AND "anti"[All Fields] AND "thymocyte"[All Fields] AND "globulin"[All Fields]))) AND ("equines"[All Fields] OR "horses"[MeSH Terms] OR "horses"[All Fields] OR "equine"[All Fields])) OR ("antilymphocyte serum"[MeSH Terms] OR ("antilymphocyte"[All Fields] AND "serum"[All Fields]) OR "antilymphocyte serum"[All Fields] OR ("lymphocytotoxic"[All Fields] AND "antibodies"[All Fields]) OR "lymphocytotoxic antibodies"[All Fields]) OR ("antilymphocyte serum"[MeSH Terms] OR ("antilymphocyte"[All Fields] AND "serum"[All Fields]) OR "antilymphocyte serum"[All Fields] OR ("antibodies"[All Fields] AND "lymphocytotoxic"[All Fields]) OR "antibodies lymphocytotoxic"[All Fields]) OR ("antilymphocyte serum"[MeSH Terms] OR ("antilymphocyte"[All Fields] AND "serum"[All Fields]) OR "antilymphocyte serum"[All Fields] OR ("antibody"[All Fields] AND "lymphocytotoxic"[All Fields])) OR ("antilymphocyte serum"[MeSH Terms] OR ("antilymphocyte"[All Fields] AND "serum"[All Fields]) OR "antilymphocyte serum"[All Fields] OR ("lymphocytotoxic"[All Fields] AND "antibody"[All Fields]) OR "lymphocytotoxic antibody"[All Fields]) OR ("antilymphocyte serum"[MeSH Terms] OR ("antilymphocyte"[All Fields] AND "serum"[All Fields]) OR "antilymphocyte serum"[All Fields] OR ("antilymphoblast"[All Fields] AND "globulins"[All Fields]) OR "antilymphoblast globulins"[All Fields]) OR ("antilymphocyte serum"[MeSH Terms] OR ("antilymphocyte"[All Fields] AND "serum"[All Fields]) OR "antilymphocyte serum"[All Fields] OR ("antilymphoblast"[All Fields] AND "globulin"[All Fields]) OR "antilymphoblast globulin"[All Fields]) OR ("antilymphocyte serum"[MeSH Terms] OR ("antilymphocyte"[All Fields] AND "serum"[All Fields]) OR "antilymphocyte serum"[All Fields] OR ("globulin"[All Fields] AND "antilymphoblast"[All Fields])) OR ("antilymphocyte serum"[MeSH Terms] OR ("antilymphocyte"[All Fields] AND "serum"[All Fields]) OR "antilymphocyte serum"[All Fields] OR ("globulins"[All Fields] AND "antilymphoblast"[All Fields]) OR "globulins antilymphoblast"[All Fields]) OR ("antilymphocyte serum"[MeSH Terms] OR ("antilymphocyte"[All Fields] AND "serum"[All Fields]) OR "antilymphocyte serum"[All Fields] OR ("antilymphocyte"[All Fields] AND "immunoglobulin"[All Fields]) OR "antilymphocyte immunoglobulin"[All Fields]) OR ("antilymphocyte serum"[MeSH Terms] OR ("antilymphocyte"[All Fields] AND "serum"[All Fields]) OR "antilymphocyte serum"[All Fields] OR ("antilymphocyte"[All Fields] AND "immunoglobulins"[All Fields]) OR "antilymphocyte immunoglobulins"[All Fields]) OR ("antilymphocyte serum"[MeSH Terms] OR ("antilymphocyte"[All Fields] AND "serum"[All Fields]) OR "antilymphocyte serum"[All Fields] OR ("immunoglobulin"[All Fields] AND "antilymphocyte"[All Fields]) OR "immunoglobulin antilymphocyte"[All Fields]) OR ("antilymphocyte serum"[MeSH Terms] OR ("antilymphocyte"[All Fields] AND "serum"[All Fields]) OR "antilymphocyte serum"[All Fields] OR ("immunoglobulins"[All Fields] AND "antilymphocyte"[All Fields]))) AND ("graft vs host disease"[MeSH Terms] OR ("graft"[All Fields] AND "vs"[All Fields] AND "host"[All Fields] AND "disease"[All Fields]) OR "graft vs host disease"[All Fields] OR ("graft"[All Fields] AND "versus"[All Fields] AND "host"[All Fields] AND "disease"[All Fields]) OR "graft versus host disease"[All Fields] OR ("graft vs host disease"[MeSH Terms] OR ("graft"[All Fields] AND "vs"[All Fields] AND "host"[All Fields] AND "disease"[All Fields]) OR "graft vs host disease"[All Fields] OR ("disease"[All Fields] AND "graft"[All Fields] AND "versus"[All Fields] AND "host"[All Fields]) OR "disease graft versus host"[All Fields]) OR ("graft vs host disease"[MeSH Terms] OR ("graft"[All Fields] AND "vs"[All Fields] AND "host"[All Fields] AND "disease"[All Fields]) OR "graft vs host disease"[All Fields] OR ("diseases"[All Fields] AND "graft"[All Fields] AND "versus"[All Fields] AND "host"[All Fields]) OR "diseases graft versus host"[All Fields]) OR ("graft vs host disease"[MeSH Terms] OR ("graft"[All Fields] AND "vs"[All Fields] AND "host"[All Fields] AND "disease"[All Fields]) OR "graft vs host disease"[All Fields] OR ("graft"[All Fields] AND "versus"[All Fields] AND "host"[All Fields] AND "disease"[All Fields]) OR "graft versus host disease"[All Fields]) OR ("graft vs host disease"[MeSH Terms] OR ("graft"[All Fields] AND "vs"[All Fields] AND "host"[All Fields] AND "disease"[All Fields]) OR "graft vs host disease"[All Fields] OR ("graft"[All Fields] AND "versus"[All Fields] AND "host"[All Fields] AND "diseases"[All Fields]) OR "graft versus host diseases"[All Fields]) OR ("graft vs host disease"[MeSH Terms] OR ("graft"[All Fields] AND "vs"[All Fields] AND "host"[All Fields] AND "disease"[All Fields]) OR "graft vs host disease"[All Fields] OR ("runt"[All Fields] AND "disease"[All Fields]) OR "runt disease"[All Fields]) OR ("graft vs host disease"[MeSH Terms] OR ("graft"[All Fields] AND "vs"[All Fields] AND "host"[All Fields] AND "disease"[All Fields]) OR "graft vs host disease"[All Fields] OR ("disease"[All Fields] AND "runt"[All Fields]) OR "disease runt"[All Fields]) OR ("graft vs host disease"[MeSH Terms] OR ("graft"[All Fields] AND "vs"[All Fields] AND "host"[All Fields] AND "disease"[All Fields]) OR "graft vs host disease"[All Fields]) OR ("graft vs host disease"[MeSH Terms] OR ("graft"[All Fields] AND "vs"[All Fields] AND "host"[All Fields] AND "disease"[All Fields]) OR "graft vs host disease"[All Fields] OR ("disease"[All Fields] AND "graft"[All Fields] AND "vs"[All Fields] AND "host"[All Fields]) OR "disease graft vs host"[All Fields]) OR ("graft vs host disease"[MeSH Terms] OR ("graft"[All Fields] AND "vs"[All Fields] AND "host"[All Fields] AND "disease"[All Fields]) OR "graft vs host disease"[All Fields] OR ("diseases"[All Fields] AND "graft"[All Fields] AND "vs"[All Fields] AND "host"[All Fields]) OR "diseases graft vs host"[All Fields]) OR ("graft vs host disease"[MeSH Terms] OR ("graft"[All Fields] AND "vs"[All Fields] AND "host"[All Fields] AND "disease"[All Fields]) OR "graft vs host disease"[All Fields] OR ("graft"[All Fields] AND "vs"[All Fields] AND "host"[All Fields] AND "diseases"[All Fields]) OR "graft vs host diseases"[All Fields]) OR ("graft vs host disease"[MeSH Terms] OR ("graft"[All Fields] AND "vs"[All Fields] AND "host"[All Fields] AND "disease"[All Fields]) OR "graft vs host disease"[All Fields] OR ("homologous"[All Fields] AND "wasting"[All Fields] AND "disease"[All Fields]) OR "homologous wasting disease"[All Fields]) OR ("graft vs host disease"[MeSH Terms] OR ("graft"[All Fields] AND "vs"[All Fields] AND "host"[All Fields] AND "disease"[All Fields]) OR "graft vs host disease"[All Fields] OR ("disease"[All Fields] AND "homologous"[All Fields] AND "wasting"[All Fields]))) AND ("transplantation, homologous"[MeSH Terms] OR ("transplantation"[All Fields] AND "homologous"[All Fields]) OR "homologous transplantation"[All Fields] OR ("allogeneic"[All Fields] AND "transplantation"[All Fields]) OR "allogeneic transplantation"[All Fields] OR ("transplantation, homologous"[MeSH Terms] OR ("transplantation"[All Fields] AND "homologous"[All Fields]) OR "homologous transplantation"[All Fields] OR ("transplantation"[All Fields] AND "allogeneic"[All Fields]) OR "transplantation allogeneic"[All Fields]) OR ("homografted"[All Fields] OR "transplantation, homologous"[MeSH Terms] OR ("transplantation"[All Fields] AND "homologous"[All Fields]) OR "homologous transplantation"[All Fields] OR "homografting"[All Fields]) OR ("transplantation, homologous"[MeSH Terms] OR ("transplantation"[All Fields] AND "homologous"[All Fields]) OR "homologous transplantation"[All Fields] OR ("homologous"[All Fields] AND "transplantation"[All Fields])) OR ("transplantation, homologous"[MeSH Terms] OR ("transplantation"[All Fields] AND "homologous"[All Fields]) OR "homologous transplantation"[All Fields] OR ("allogeneic"[All Fields] AND "grafting"[All Fields]) OR "allogeneic grafting"[All Fields]) OR ("transplantation, homologous"[MeSH Terms] OR ("transplantation"[All Fields] AND "homologous"[All Fields]) OR "homologous transplantation"[All Fields] OR ("grafting"[All Fields] AND "allogeneic"[All Fields]) OR "grafting allogeneic"[All Fields]) OR ("allograft s"[All Fields] OR "allografted"[All Fields] OR "allografts"[MeSH Terms] OR "allografts"[All Fields] OR "allograft"[All Fields] OR "transplantation, homologous"[MeSH Terms] OR ("transplantation"[All Fields] AND "homologous"[All Fields]) OR "homologous transplantation"[All Fields] OR "allografting"[All Fields]))
